# Double-Gene Copromoting Expression Analysis in tPA/GH Transgenic Goat Mammary Epithelial Cells and Thrombolytic Activity of tPA In Vitro

**DOI:** 10.1155/2022/6484073

**Published:** 2022-05-06

**Authors:** Shaozheng Song, Yaoling Luo, Zhaoxia Liu, Dan Li, Junsong Ye, ZhengYi He

**Affiliations:** ^1^School of Health and Nursing/Department of Basic, Wuxi Taihu University, China; ^2^Department of Clinical Medicine Research Center, First Affiliated Hospital of Gannan Medical University, Ganzhou, Jiangxi, China; ^3^Department of Reproductive Medicine Research Center, First Affiliated Hospital of Gannan Medical University, Ganzhou, Jiangxi, China; ^4^Subcenter for Stem Cell Clinical Translation, First Affiliated Hospital of Gannan Medical University, Ganzhou, Jiangxi, China; ^5^Ganzhou Key Laboratory of Stem Cell and Regenerative Medicine, Ganzhou, Jiangxi, China; ^6^Key Laboratory of Prevention and Treatment of Cardiovascular and Cerebrovascular Diseases, Ministry of Education, Gannan Medical University, Ganzhou, Jiangxi, China; ^7^Key Laboratory of Biomaterials and Biofabrication in Tissue Engineering of Jiangxi Province, Gannan Medical University, Ganzhou, Jiangxi, China

## Abstract

Human tissue-plasminogen activator (tPA) is a thrombolytic drug widely used in the treatment of stroke, pulmonary thrombosis, acute myocardial infarction, and other thrombotic diseases. The double genes cointegrated into the organisms and cells can produce a synergistic effect, which will improve the expression level of the target gene. However, the study of the integration of the GH and tPA genes to improve the expression level of tPA has not yet been reported. In order to elucidate this, we generated monoclonal goat mammary epithelial cell lines with tPA/GH double-gene integration and analyzed the tPA expression level in single- and double-gene integrated cells. We selected the mammary gland-specific expressing vectors BLC14/tPA and BLC14/GH with the *β*-lactoglobulin gene as a regulatory sequence in our previous research. The tPA and GH genes were electronically cotransfected into goat mammary epithelial cells. Resistant cell lines were screened by G418, and transgenic monoclonal cell lines were confirmed by PCR. The tPA expression was induced by prolactin and detected in the cell induction solution after 48 h by ELISA and Western blotting. We detected the tPA biological activity in vitro by fibrin agarose plate assay (FAPA). The results showed that a total of 207 resistant monoclonal cells were obtained, including 126 cell lines with tPA monogenic integration and 51 cell lines with tPA/GH double-gene integration. The rate of double-gene integration was 24.6% (51/207). A total of 48 cells expressed tPA, of which 25.3% (19/75) cells expressed single gene, and 56.9% (29/51) cells expressed double genes. The concentration of tPA in single-gene-expressing cells was 8.0-64.0 *μ*g/mL, and the tPA level in double-gene-expressing cells was significantly higher (200-7200 *μ*g/mL). In addition, the tPA had a relatively strong in vitro thrombolytic activity determined by FAPA. The results showed that goat mammary epithelial cell lines with tPA/GH gene integration were successfully established by electrotransfection, and the expression level of tPA in double-gene integrated cell lines was significantly increased. This study provided a new way for the preparation of a transgenic goat and other animal with high tPA expression by somatic cell nuclear transfer. The findings also laid a foundation for efficient production of pharmaceutical proteins in transgenic animal mammary gland bioreactors in the future.

## 1. Introduction

Since the 20th century, cardiovascular disease has become a leading cause of death in humans, and its pathogenesis is commonly related with the thromboembolic condition. Currently, thrombolytic therapy is the most effective treatment in managing thromboembolism [1–3]. Human tissue-type plasminogen activator (tPA) is a serine protease synthesized and secreted by vascular endothelial cells. As an effective thrombolytic drug, it can efficiently dissolve thrombus [4]. At present, the clinically used thrombolytic drugs are mainly produced by overexpressing proteins in engineered bacteria such as Escherichia coli, and this method shows disadvantages such as low yield, low activity, poor compatibility with human body, and high price, which is not conducive to popularization and application of the drugs.

In 1987, Gordon et al. generated the first transgenic mice that expressed tPA in the mammary gland [4], and many other reports elucidated the expression of tPA in mouse mammary gland [5, 6]. Lu et al. reported a tPA level of 6 *μ*g/mL in the mammary gland of transgenic mice [5]. Zhou et al. reported the highest level of tPA (3.3 g/L) in transgenic mouse mammary gland [6]. In 1991, Ebert et al. first reported a tPA level of 3 *μ*g/mL in transgenic goat mammary gland [7]. In 2015, we investigated the expression of tPA in goat mammary epithelial cells, but the expression of tPA was shown to be relatively low [8]. In addition, goat growth hormone (GH, the “GH” is the acronym for growth hormone) is a protein secreted by the anterior pituitary, which shares a similar structure to prolactin and controls the activation of*α*s-casein and *β*-casein. GH promotes the growth and development of the mammary gland and maintains lactation [9, 10].

It has been proved that the transgenic organism or cells that have double-gene cointegration can produce a synergistic effect and improve the expression level of the target gene [11]. Zhao et al. [12] showed that through adenovirus-mediated ING4/IL-24 dual tumor suppressor gene cotransfer, the synergistic promotion of double-gene coexpression in human mammary cancer cells enhanced the antitumor activity of Ad-ING4-IL-24. Park et al. [13] successfully generated the DM1919 strain by targeted modification of sgRNA-gltA-r citrate synthase- and used CRISPRi to mediate double-gene transfer of idsA and glgC into Corynebacterium glutamicum to increase L-lysine expression. Sendtner et al. [14] found that the expression level of leukemia inhibitory factor (LIF) was significantly increased following the transfection of ciliary neurotrophic factor (CNTF) and LIF genes to improve the nutritional and physiological effects on motorneurons. However, there have no reports about the cointegration effect of tPA and GH genes transfected into organisms or cells on improving the expression of tPA, especially in goat mammary epithelial cells.

In addition, the development of animal mammary gland bioreactors may face many problems, such as a long development cycle, huge initial investment, many technical difficulties, and a complex regulatory mechanism underlying milk protein expression specificity [15]. Therefore, it is very important to study protein expression at the cellular level. Goat mammary epithelial cells can be induced to express specific proteins, which provides a good tool for early cell-level validation studies [8]. In this study, BLC14/tPA and BLC14/GH mammary gland specific-expression vectors with *β*-lactoglobulin gene as the regulatory sequence were used. The tPA and GH genes were cotransfected into goat mammary epithelial cells to elucidate the effect of dual-gene cointegration on tPA expression, which provided a new idea and method for preparing transgenic animals that highly express tPA protein by somatic cell nuclear transfer. This study is aimed at establishing transgenic animal mammary gland bioreactors for the production of pharmaceutical proteins using a novel technical approach.

## 2. Materials and Methods

### 2.1. Materials

**Figure 1:**
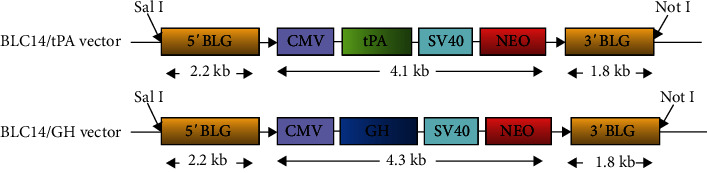
Gene structure of mammary gland-specific expression vectors BLC14/tPA and BLC14/GH.

Saanen dairy goats in the middle and late stages of lactations were routinely raised in the experimental farm of Yangzhou University. The mammary gland-specific expression vectors, BLC14/tPA and BLC14/GH, were constructed and preserved in our laboratory (Figure 1). The tPA and GH cDNAs were obtained. BLC14 was a mammalian expression vector containing a regulatory element of goat *β*-lactoglobulin and a CMV promoter, which were confirmed in cultured goat cells, mice, and rabbits [8, 15]. The lactoglobulin mRNA 5′UTR and downstream 3′UTR were designed based on the published sequences in GeneBank (NCBI, Z33881.1, 8,088 bp), and the corresponding sequence loci on the genome were -3528~-1331, respectively.

### 2.2. Design and Synthesis of Primers

The PCR primers were designed using the software Primer Premier 5.0, and the primers were synthesized by Shanghai Sangon Bioengineering Technology Co., Ltd. (Shanghai, China) (Table 1).

### 2.3. Separation and Purification of Goat Mammary Epithelial Cells

Approximately 5 g of lactating goat mammary gland parenchyma was removed by aseptic surgery and washed repeatedly with D-hank's buffer solution until it was free of blood and milk. The acinar tissue was cut into pieces with a size of about 1 mm^3^, and 30 mL collagenase type I digestion solution (with 200 IU/mL collagenase type I and 100 IU/mL hyaluronidase) was added for digestion reaction. The tissues were incubated on a shaker for 1 h. Cell suspensions were collected and filtered using a 300-mesh screen. The filtrate was centrifuged and washed three times with D-hank's buffer. The cells were counted, and the cell density was adjusted to 1 × 10^6^ cells/mL with culture medium (DMEM/F12, Thermo Fisher Scientific; 10% FBS, Hyclone Laboratories; 5 mM sodium acetate, 5 *μ*g/mL transferrin, 0.5 mM cholamine, 10 *μ*g/mL insulin, and 5 *μ*g/mL hydrocortisone, Sigma-Aldrich). Cells were seeded into a six-well plate and placed in a CO2 incubator at 37°C, with 5% CO2 and saturated humidity for stationary cultivation. After reaching 80% confluence, the adherent cell layer was digested with 0.05% trypsin +0.04% EDTA for 2 min, and the digested fluid containing desorbed fibroblasts was discarded. The cell mixture was seeded into the plate, and the culture medium was added to regrow cells. Pure mammary epithelial cells were obtained after repeating this procedure twice.

### 2.4. Cotransfection of BLC14/tPA and BLC14/GH into Goat Mammary Epithelial Cells

BLC14/tPA and BLC14/GH vectors were linearized by Sal I and Not I double-enzyme digestion, recovered using a kit from Qiagen, dissolved in ultrapure water, and stored at -20°C. The goat mammary epithelial cells were collected during the logarithmic growth phase, washed, and centrifuged using electrotransfection fluid. The cells were resuspended to a density of 1 × 10^6^/mL, and the DNA plasmids were added (final concentration of 20 *μ*g/mL). Cells were electrically shocked once at 2.0 KV/cm for 250 *μ*s (Multiporator electroporation instrument; Eppendorf, Hamburg, Germany). After 48 h, 500 *μ*g/mL G418 (Thermo Fisher Scientific, Eugene, OR, USA) was added for screening, and the liquid was changed every two days. After 10-14 days, the cloned cells were selected and multiplied on 48-well and 12-well culture plates. Partial cells were collected and cryopreserved with DMEM/F12 +10% DMSO +20% FBS, and the remaining cells were used for subsequent experiments. Cell passage calculation was as follows: F0 generation was isolated from goat mammary epithelial cells. After screening and culturing BLC14/tPA and BLC14/GH positive cells, the monoclonal cells were selected for extended culturing, and about 2-3 cell generations (F3-F5 or F4-F6 generation) were transferred. According to our previous SCNT studies, the fewer cell passages were, the stronger the cell vitality became, and the higher the efficiency of nuclear transfer cloning would reach. However, after more than 10 generations of transfer, the cells could also successfully clone animals through nuclear transfer.

### 2.5. PCR Detection of Monoclonal Cell Lines

Partial cell suspensions were collected after digesting with 0.05% skin protease and 0.04% EDTA and centrifuged at 2000 rpm for 5 min. Then, the supernatant was discarded, and the cells were resuspended with 10 *μ*L cell lysate. After incubation at 45°C for 45 min and 96°C for 15 min, the cells were directly used as PCR templates for tPA and GH gene integration detection. The tPA-F/R primers were used for tPA gene integration detection. The PCR amplification conditions were as follows: predenaturation at 95°C for 5 min; 30 cycles of denaturation at 94°C for 45 sec, annealing at 45°C for 35 sec, and elongation at 72°C for 45 sec; and stopping the reaction and storing at 4°C. GH-F/R primers were used to detect GH gene integration. The PCR amplification conditions were as follows: predenaturation at 95°C for 5 min; 30 cycles of denaturation at 95°C for 45 sec, annealing at 52°C for 45 sec, and extension at 72°C for 1 min; and stopping the reaction and storing at 4°C. Primer sequences are shown in Table 1.

### 2.6. Enzyme-Linked Immunosorbent Assay (ELISA)

When the cells cultured in a 12-well plate reached about 80% confluency, the old culture medium was discarded, and the fresh culture medium containing 5 *μ*M prolactin was added for inducing protein expression. After 48 h, the cell induction liquid was collected for ELSA analysis. Mouse anti-tPA monoclonal antibody (sc-59721; Santa Cruz Biotechnology, Santa Cruz, CA, USA) was used as primary antibody, and the sheep anti-mouse monoclonal antibody IgG-HRP was used as a secondary antibody (sc-2005; Santa Cruz Biotechnology). The OD450 values were determined. A standard curve was constructed, and the tPA expression was calculated and compared with its expression levels induced by tPA/gGH double-gene integration and tPA single-gene integration in goat mammary epithelial cells. The tPA reference materials used for Western blotting analysis and ELISA were purchased from the National Institute for Biological Standards and Control (NIBSC, Potters Bar Britain). The ELISA procedure was as follows:

A total of 100 *μ*l sample was 1 : 1 diluted by coating solution, added to each well of the 96-well plate, and incubated at 4°C for 24 h. Nontransgenic mammary epithelial cell culture was simultaneously induced, and the supernatant was used as negative control. Sterilized double steaming water was used as blank control, and alteplase thrombolytic drug was used as positive control. The coating solution was discarded, the plate was washed 3 times with PBS-T, 5 min each time, then 200 *μ*L blocking solution (PBS containing 10% calf serum) was added to each well, and the plate was incubated at 37°C for 2 h. The sealing solution was discarded, the plate were washed 3 times with PBS-T. Then 100 *μ*L diluted primary tPA antibody (1 : 2000 diluted, Santa Cruz) was added to each well, and the plate was incubated at 37°C for 2 h. After washing with PBS-T for 3 times, 100 *μ*L diluted HRP-conjugated secondary antibody (1 : 2000 diluted, Goat Anti-Mouse IgG Monoclonal Antibody, Santa Cruz) was added to each well, and the plate was incubated at 37°C for 2 h. After washing with PBS-T, 50 *μ*L chromogenic solution (10 mL enzyme substrate buffer +5 mg OPD +15 *μ*L 30% H2O2, ready for use) was added to each well, and the plate was incubated at 37°C for 15 min, avoiding light. Then, 50 *μ*L 2 M H_2_SO_4_ was added to each well to stop color development. The value of OD450 and the judgment result were measured with a RT-6000 type microplate analyzer, and the photos were recorded and saved.

The tPA (purchased from NIBSC) was used as the standard.

### 2.7. Western Blotting

A 12% SDS-PAGE electrophoresis was performed [15]. The proteins were transferred to a PVDF membrane using the transfer buffer (1.93 g/L tris and 9 g/L glycine) at 250 mA for 3.5 h. After washing with ultrapure water, the membrane was blocked at 37°C for 2.5 h in a blocking buffer containing 20 mM Tris, 137 mM NaCl, 0.1% Tween-20, and 10% fetal bovine serum (pH 7.6). The membrane was incubated with the diluted mouse anti-tPA monoclonal primary antibody (1 : 2000; sc-59721, Santa Cruz Biotechnology) for 2 h at 37°C. After washing with TTBS (20 mM Tris, 137 mM NaCl, 1% Tween-20, and pH 7.6) three times, the membrane was incubated with the diluted sheep anti-mouse monoclonal secondary antibody (1 : 2000; IgG-HRP; sc-2005, Santa Cruz Biotechnology) for 2 h at 37°C. Finally, the PVDF membrane was washed with PBS, and the color-substrate solution (DAB 50 mg, 0.05 mol/L TB100 mL, 30 *μ*L 30% H2O2, and pH 7.6) was added for 15 min at room temperature; then, the images were taken, and proteins were quantified.

### 2.8. In Vitro Characterization of tPA Thrombolytic Activity in Transgenic Goat Mammary Epithelial Cell Induction Liquid

In vitro thrombolytic activity of rhPA in transgenic goat mammary epithelial cell induction liquid was evaluated by fibrin agarose plate assay (FAPA) [15]. The tPA transgenic goat mammary epithelial cell induction liquids were diluted with PBS. The fibrinogen-thrombin-agarose gel plate consisting of 1.0% (w/v) agarose, 10 mg/mL fibrinogen, and 1 U/mL thrombin in PBS (137 mM NaCl, 10 mM Na2HPO4, 3 mM KCl, 2 mM KH2PO4, and pH 7.4) was prepared. The agarose gel (20 mL) was boiled and melted completely. Fibrinogen solution (1 ml) was preheated at 37°C, and thrombin was prewarmed to 42°C. When the temperature of the gel was dropped to 55-60°C, fibrinogen and thrombin were added, mixed with the gel rapidly, and the gel was poured into a 10 × 15 cm^2^ rectangular transparent plexiglass plate. When the solution was cooled to room temperature, the sample wells were drilled on the gel plate. Finally, each sample well was filled with 20 *μ*L sample solution and incubated at 37°C for 12 h. The tPA thrombolytic activity was determined in vitro by the size of the transparent zone of the thrombin-dissolving ring.

## 3. Results and Analysis

### 3.1. Morphological Structure of Goat Mammary Epithelial Cells

**Figure 2:**
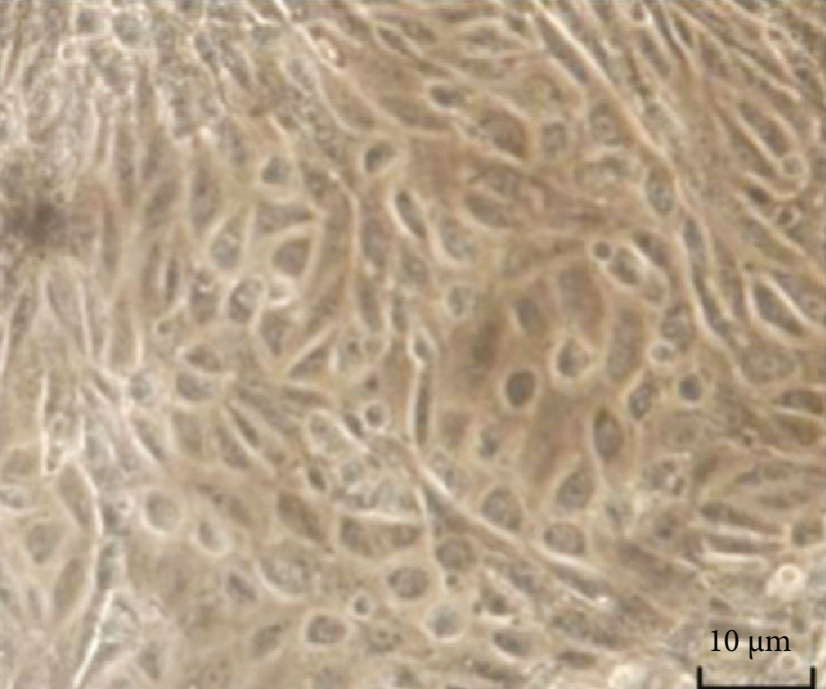
Typical morphological structure of goat mammary epithelial cells (100×).

Goat mammary epithelial cells were isolated by collagenase digestion and cultured in vitro grew vigorously and homogeneously, presenting honeycomb or island-like morphology with clear boundaries. Unpurified mammary epithelial cells were occasionally mixed with fine fibroblasts. Typical goat mammary epithelial cells were large adherent cells with a short fusiform, polygonal, round, or cobblestone appearance (Figure 2).

### 3.2. PCR Integration Detection

**Figure 3:**
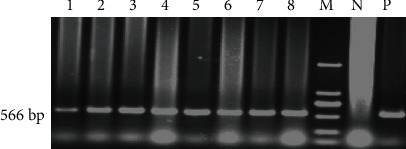
Detection of transfected tPA gene in goat mammary epithelial cells by PCR. 1-8: monoclonal mammary epithelial cell DNA; M: DL2000 digested DNA Marker; N: negative control (normal cell genome); P: positive control (BLC14/tPA plasmid).

**Figure 4:**
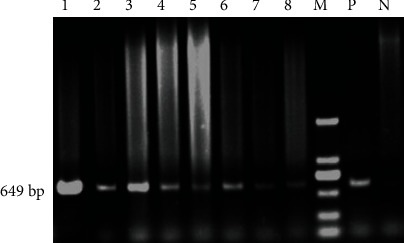
Detection of transfected GH gene in goat mammary epithelial cells by PCR. 1-8: monoclonal mammary epithelial cell DNA; M: DL2000 digested DNA Marker; P: positive control (BLC14/GH plasmid); N: negative control (normal cell genome).

A total of 207 monoclonal resistant cells was obtained. The tPA-F/R primers were used for PCR amplification to identify 126 strains with integrated exogenous tPA genes, and the results illustrated a 566 bp target band (Figure 3). The GH-F/R primers were used to detect GH gene integration in these 126 cells, and a total of 51 GH gene integration cell lines were identified through the detection of a target band with 649 bp (Figure 4). There were 75 cell lines without GH gene integration. In other words, a total of 75 mammary epithelial cell lines with tPA single-gene integration were obtained, with an integration efficiency of 36.2% (75/207). A total of 51 strains of tPA/GH double-gene integrated mammary epithelial cell lines were obtained, with an integration efficiency of 24.6% (51/207), as detailed in Table 2.

### 3.3. Induction of tPA Expression in Monoclonal Cell Lines

**Figure 5:**
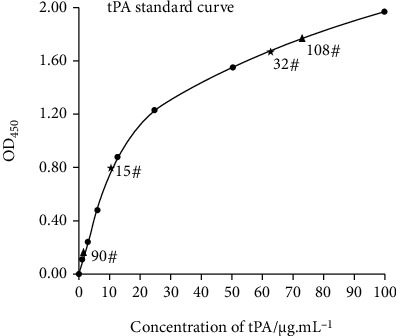
Detection of the tPA expression in transgenic monoclonal mammary epithelial cell lines. The concentrations of the tPA standard are 0, 1.5625, 3.125, 6.25, 12.5, 25, 50, and 100 *μ*g/mL, respectively; 15 # and 32 #, respectively, show the highest and lowest level of tPA expression induced in goat mammary epithelial cell lines with tPA single-gene integration; 90# and 108# (diluted 100 times), respectively, show the highest and lowest level of tPA expression induced in goat mammary epithelial cell lines with tPA/GH double-gene integration.

The tPA expression was induced by prolactin in goat mammary epithelial cells containing tPA single gene and tPA/GH double genes, and the cell fluid after 48 h induction was collected for ELISA detection. The experiment was repeated once, and the average value of the results was used. A total of 48 cells expressed tPA, with an expression rate of 38.1% (48/126), including 19 single-gene-expressing cells with an expression rate of 25.3% (19/75) and 29 double-gene-expressing cells with an expression rate of 56.9% (29/51), as shown in Table 2. The standard curve was constructed with different concentrations (*μ*g/mL) of the tPA standard substance (alteplase) as the abscissa and the value of OD450 as the ordinate (Figure 5). The tPA level in goat mammary gland cells with single-gene integration was 8.0-64.0 *μ*g/mL, while cells with double-gene integration showed a significantly higher value of 200-7200 *μ*g/mL.

### 3.4. Western Blotting Detection

**Figure 6:**
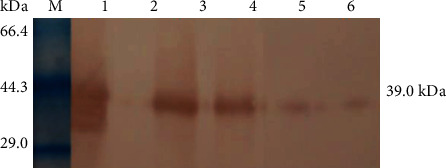
Western blotting detection of tPA expression in transgenic monoclonal mammary epithelial cell lines. M: protein molecular weight standard; 1: positive control (tPA standard); 2: negative control (normal nontransgenic cell inducer); 3-6: expression of cell inducer (108#, 90#, 32#, and 15#).

Western blotting detection results of the proteins in induction fluids are shown in Figure 6. The tPA-expressing cell induction fluid and positive control (tPA standard substance) produced a band with a size of about 39.0 kDa, while no such band was found from the tPA-nonexpressing cell induction fluid and the negative control. The results preliminarily determined that prolactin induced tPA expression in goat mammary epithelial cells.

### 3.5. Biological Activity of tPA in Transgenic Goat Mammary Epithelial Cell Induction Liquid

**Figure 7:**
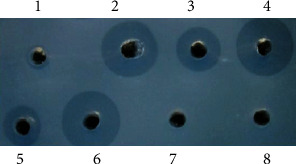
FAPA of goat mammary epithelial cell induction solution. 1: 1.0 *μ*g/mL alteplase standard; 2: 100 *μ*g/mL alteplase standard; 3: 90# tPA/GH double transgenic goat mammary epithelial cell induction liquid (diluted 100 times); 4: 108# tPA/GH double transgenic goat mammary epithelial cell induction liquid (diluted 1000 times); 5: 15# tPA single transgenic goat mammary epithelial cell induction fluid (diluted 10 times); 6: 32# tPA single transgenic goat mammary epithelial cell induction fluid (diluted 10 times); 7: normal nontransgenic goat mammary epithelial cell induction fluid; 8: PBS.

The tPA is a serine protease that converts plasminogen into active plasmin, which digests fibrin and dissolves fibrin clots. In this study, the activity of tPA in transgenic goat mammary epithelial cell induction liquid was detected by FAPA. The fibrinolytic activity of the tPA was determined according to the size of the thrombolytic transparent circle. FAPA analysis showed that the tPA produced by transgenic goat mammary epithelial cells had higher fibrinolytic activity in vitro compared with the alteplase standard products (Figure 7). Furthermore, the thrombolytic transparent circles were not observed in normal nontransgenic goat mammary epithelial cell induction liquid and PBS.

## 4. Discussion

With the continuous change in people's living standards, dietary structure, and living habits, the cardiovascular diseases in China, especially those with thrombotic conditions, tend to develop in young people, and their incidence has shown an upward trend [16–18]. Human tissue-type plasminogen activator (tPA) is an important thrombolytic drug for the treatment of thrombotic diseases [18]. However, how to efficiently, cheaply, and easily produce tPA has puzzled the scientific community for a long time. Wright et al. successfully expressed human *α*-antitrypsin gene in goat mammary gland in the 1990s. Since then, the development of mammary gland bioreactor has made great progress, which provides a great possibility for the production of tPA thrombolytic drugs [19]. Animal mammary gland bioreactor can produce high-yield thrombolytic drugs, such as tPA, with low-cost posttranslational modification and higher biological activity compared with the other expression systems [8]. However, the expression level of tPA in the mammalian mammary gland is low, which may be due to the fact that the tPA is a nonmilk protein, the mechanism underlying milk protein-specific expression is complex, and the gene network involves the interaction among multiple genes and regulatory elements. Therefore, it is very important to explore how to enhance the expression of exogenous tPA in mammalian mammary gland.

Cointegration of two genes into animals will produce synergistic effects [20, 21]. One gene presence can promote the expression level of the other target gene, which helps to solve the problem of low tPA production in transgenic animals, achieving higher yield. In recent years, it has been reported that double-gene integration may improve the expression level of the exogeneous target gene. For example, Domingues et al. cloned the *α*-Hb and *β*-HB subunits of hemoglobin tetramer into the downstream region of Trc promoter, producing coexpression of multiple genes [11]. Guo et al. [22] constructed a recombinant adenovirus vector containing H5N1 influenza A virus M1 and HA genes and transfected this vector into 293 cells. The results showed that the M1 and HA genes were efficiently coexpressed. Sendtner et al. [14] found that upregulation of LIF significantly increased nutritional and physiological related effects on motoneurons by cotransfection of CNTF and LIF genes. Chrysovergis et al. [23] also proved that double-gene integration promoted the expression of target genes in laryngeal squamous cell carcinoma. The reason may be related to the gene regulatory network in the organism and the complex and multilevel regulation of gene expression. Some gene expressions can activate and improve the transcription and expression level of another gene [11, 24]. However, there are few reports on dual-gene integration effects in goat mammary epithelial cells, especially on the expression analysis of tPA/GH gene cotransfected goat mammary epithelial cells.

Growth hormone (GH) can promote the expression of milk protein genes [25, 26]. It can bind the HRE sequence on the milk protein gene, activate the receptor, increase the specific expression of proteins, and stimulate the differentiation and proliferation of mammary epithelial cells [27]. Therefore, goat *β*-lactoglobulin gene was selected as the regulatory sequence in this study. Mammary gland-specific expression vectors BLC14/tPA and BLC14/GH constructed and verified in the previous stage were used to realize double-gene cotransfection. However, the establishment of animal mammary gland bioreactor was associated with some problems such as a long development cycle, complex technology, high animal cost, and high risk [15]. Therefore, it is necessary to verify in the early stage of its development. Mammalian cells and mice are the most commonly used to validate the expression vectors [8, 15]. Although goat mammary epithelial cells are terminally differentiated cells that are extremely difficult to culture, their differentiation can be induced by prolactin [28, 29]. Therefore, goat mammary epithelial cells are most widely used to verify mammary gland-specific expression vectors. Furthermore, the GH gene selected in this study might promote the growth of mammary epithelial cells, as evidenced by many transgenic cell lines successfully obtained in this study.

In this study, both BLC14/tPA and BLC14/GH gene vectors were electrically cotransfected with the *β*-lactoglobulin gene, which acted as the regulatory sequence. A total of 51 transgenic mammary epithelial cells with tPA/GH dual-gene integration was obtained, with an integration rate of 24.6%, while 75 cells with tPA single-gene integration were obtained, showing a 36.2% integration rate. The transgenic integration efficiency was high. The results of prolactin-induced expression showed that double-gene integration significantly improved the expression of exogenous tPA target gene in transgenic cells, with the highest expression level of 7200 *μ*g/mL. The expression rate was also increased, significantly higher than that of single-gene integrated cell line (56.9% vs. 25.3%). The reason may be that goat GH gene played a synergistic promoting role with goat milk protein gene regulatory sequence, thus improving the expression levels of exogenous tPA gene. At the same time, due to the low level of single-gene expression, some cell lines expressed very low level of tPA, which made it not easy to be detected by ELISA. When an additional goat GH gene with a synergistic promoting effect on expression was transferred, the level of tPA in some cell lines with very low original expression was significantly increased and tPA could be detected by ELISA. Therefore, the expression rate of the tPA/GH double-gene integrated cells was significantly higher than that of tPA single-gene integrated cell lines. Moreover, we also utilized the FAPA method to detect the thrombolytic biological activity of the expressed tPA in vitro and found that its thrombolytic biological activity was much higher than that of the alteplase standard control product.

However, the expression of the genes involved in the integration site is influenced by epigenetic inheritance, exogenous gene copy number, related hormone level, gene network, etc. [15, 27, 30]. Therefore, it is necessary to continue with the related follow-up research.

In conclusion, we successfully prepared the goat mammary gland epithelial cell line with tPA/GH double-gene integration and proved the feasibility of this strategy in efficient expression of exogenous target gene tPA at the cellular level. Our findings will lay a foundation for the preparation of transgenic goats and other animals that can highly express the target genes by somatic cell nuclear transfer in the future. This study may also provide a prerequisite for the establishment of transgenic animal mammary gland bioreactors to produce pharmaceutical proteins and transgenic breeding animals.

## Figures and Tables

**Table 1:** PCR primer sequences.

Primer name	Primer sequence (5′–3′)
tPA-F	CGTGGATAGCGGTTTGA
tPA-R	GAGCCCTCCTTTGATGC
GH-F	GCGGATGATGGCTGCAGGCCC
GH-R	GAGCGGCTAGAAGGCACAGCT

**Table 2:** Statistical table of goat mammary epithelial cells transfected with tPA single gene and tPA/GH double genes.

Cell (207)	Number of integrated cells	Integration rate	Number of cells expressed	Expression rate	Expression level (*μ*g/mL)
tPA single-gene integrated cell	75	36.2%	19	25.3%	8.0-64.0
tPA/GH double-gene integrated cell	51	24.6%	29	56.9%	200-7200

## Data Availability

The data used to support the findings of this study are included within the article.
